# Increased sagittal patellar height and reduced trochlear engagement on MRI are associated with recurrent patellar instability in skeletally immature knees: A comparative study

**DOI:** 10.1002/jeo2.70896

**Published:** 2026-08-21

**Authors:** Francesco Puglia, Paolo Ferrua, David H. Dejour, Marco Nicoletti, Alessio Volpe, Riccardo Compagnoni, Antonio Memeo, Pietro Simone Randelli

**Affiliations:** ^1^ U.O.C. Ortopedia e Traumatologia Pediatrica, ASST Gaetano Pini/CTO Milan Italy; ^2^ U.O.C. Ortopedia Traumatologia Week Surgery, ASST Gaetano Pini/CTO Milan Italy; ^3^ Department of Biomedical Sciences for Health University of Milan Milan Italy; ^4^ Lyon‐Ortho‐Clinic, Clinique de la Sauvegarde, Ramsay Santé Lyon France; ^5^ Orthopedics and Traumatology Residency Program University of Milan Milan Italy; ^6^ 2° Clinica Ortopedica, ASST Centro Specialistico Ortopedico Traumatologico Gaetano Pini‐CTO Milan Italy; ^7^ Department of Biomedical, Surgical and Dental Sciences University of Milan Milan Italy; ^8^ U.O.C. 1° Clinica Ortopedica, ASST Gaetano Pini/CTO Milan Italy; ^9^ Laboratory of Applied Biomechanics, Department of Biomedical Sciences for Health University of Milan Milan Italy; ^10^ Research Center for Adult and Pediatric Rheumatic Diseases (RECAP‐RD), Department of Biomedical Sciences for Health Università degli Studi di Milano Milan Italy

**Keywords:** MRI measurements, patella alta, patellar instability, patellofemoral biomechanics, pediatric knee

## Abstract

**Purpose:**

To apply the magnetic resonance imaging (MRI)‐based concept of functional patella alta to skeletally immature patients and compare sagittal patellar height and trochlear engagement between paediatric knees with objective patellar instability (OPI) and controls without patellofemoral disorders.

**Methods:**

This retrospective study included 85 skeletally immature knees (42 OPI and 43 controls) from 83 patients undergoing MRI between 2021 and 2025. OPI required at least two lateral patellar dislocations. Measurements included patellar height index (PHI), sagittal patellar cartilage length (SPCL), patello‐tibial distance (PTD), patellar tendon length (PTL), sagittal trochlear cartilage length (STCL) and sagittal patellofemoral engagement (SPE). Three blinded observers measured every knee; their mean was analysed. Functional patella alta was defined using control‐derived interquartile‐range thresholds. Interobserver reliability used two‐way random‐effects, absolute‐agreement intraclass correlation coefficients (ICCs). Group comparisons and logistic regression were performed.

**Results:**

OPI knees had significantly higher PHI (1.178 ± 0.171 vs. 1.083 ± 0.132; *p* = 0.005) and lower SPE (0.354 ± 0.207 vs. 0.484 ± 0.200; *p* = 0.004), with greater SPCL, PTD and PTL and shorter STCL (all *p* < 0.05). Groups were unmatched for age, with older OPI patients (*p* < 0.001). Functional patella alta occurred in 16/85 knees (18.8%): 14/42 OPI (33.3%) and 2/43 controls (4.7%) and was strongly associated with OPI (odds ratio [OR] 10.25; 95% confidence interval [CI] 2.16–48.66; *p* = 0.003). Reliability was excellent for SPE (ICC 0.95), PTL (0.97) and PTD (0.94), good for SPCL (0.80) and moderate for PHI (0.72).

**Conclusion:**

Paediatric recurrent patellar instability is characterised by increased patellar height and reduced sagittal trochlear engagement on MRI. Functional patella alta applies to skeletally immature knees, but instability occurs at lower sagittal engagement values than in adults. These cohort‐specific exploratory thresholds require external validation before clinical use.

**Level of Evidence:**

Level III, retrospective comparative study.

## Graphical abstract

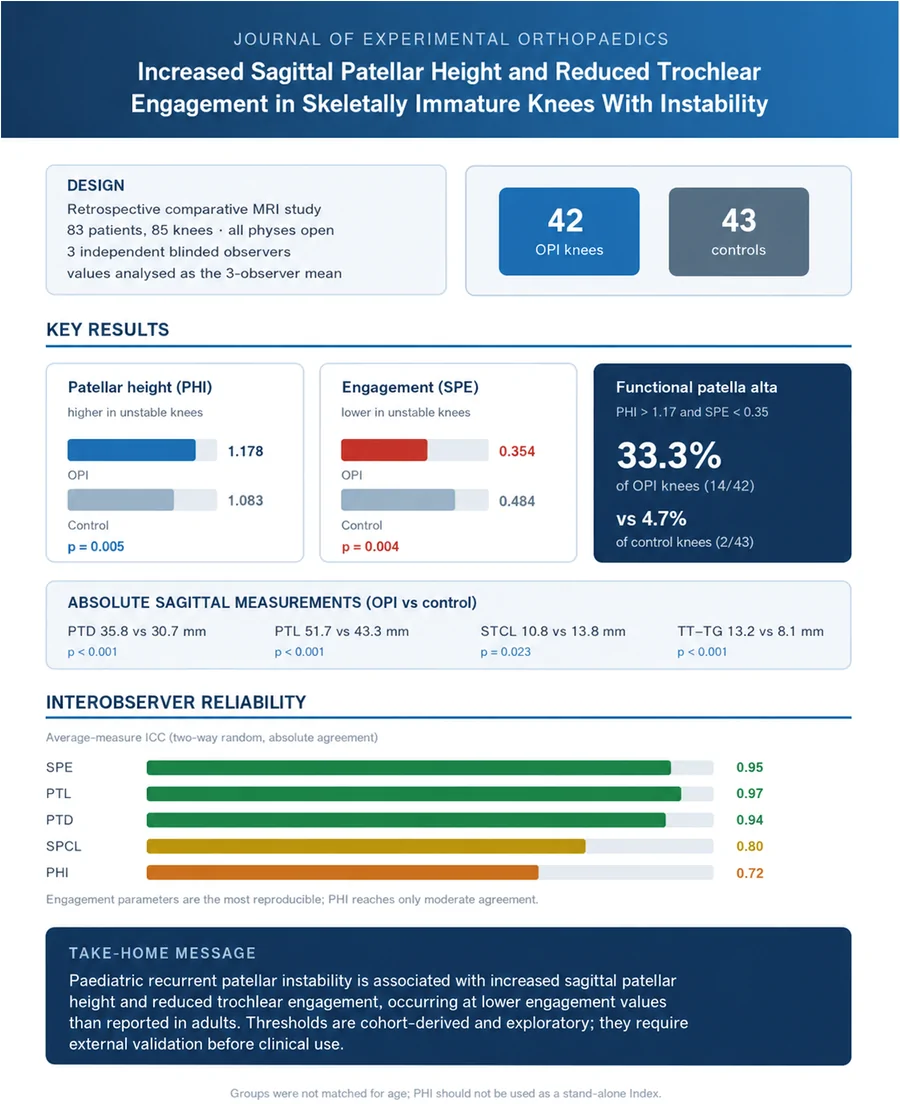

**Abbreviations**
OPI: objective patellar instability
PHI: patellar height index
PTD: patello‐tibial distance
PTL: patellar tendon length
SPCL: sagittal patellar cartilage length
SPE: sagittal patellofemoral engagement
STCL: sagittal trochlear cartilage length

## INTRODUCTION

Patellar instability is a frequent and clinically relevant cause of knee morbidity in children and adolescents [4, 8, 30, 35]. Several anatomic factors—including patella alta, trochlear dysplasia and increased tibial tubercle–trochlear groove (TT–TG) distance—have been consistently associated with lateral patellar instability [2, 9, 29]. Traditional assessment of patellar height has relied on tibial‐referenced radiographic indices such as the Insall–Salvati and Blackburne–Peel ratios [5, 17, 25, 28]. However, these tibial‐referenced indices may incompletely characterise functional patellofemoral relationships and instability‐related morphology in the paediatric population, in which continued skeletal growth modifies the reference landmarks on which they depend [20, 23, 29, 36].

Magnetic resonance imaging (MRI) enables comprehensive evaluation of cartilage morphology, trochlear anatomy and patellofemoral alignment while avoiding ionizing radiation—an important consideration in paediatric populations [18, 21]. Recent adult studies have introduced MRI‐based frameworks integrating patellar height relative to both the tibia and the femoral trochlea, defining the concept of functional patella alta and sagittal patellofemoral engagement (SPE) [10, 12]. Nevertheless, paediatric‐specific evidence for this framework remains limited, particularly because patellofemoral morphology evolves substantially during skeletal growth [14, 29, 34]. Within the paediatric and adolescent population, MRI‐based morphological analysis of the patellofemoral joint has recently been applied to both soft‐tissue and osseous structures [1, 3, 7, 14, 32, 34], while MRI‐derived quantitative criteria and cut‐off values have been proposed for individual dysplasia parameters [6, 13, 16, 33].

The purpose of this study was therefore to apply the MRI‐based concept of functional patella alta to a paediatric population and to determine whether sagittal patellar height and patellofemoral engagement differ between skeletally immature patients with objective patellar instability (OPI) and controls without patellofemoral disorders. It was hypothesised that paediatric OPI would be associated with increased patellar height and reduced sagittal engagement, with at least moderate interobserver reproducibility of the MRI‐based measurements, as reported in previous studies evaluating radiological measurements in patellofemoral instability.

## METHODS

### Study design and cohort

**Figure 1:**
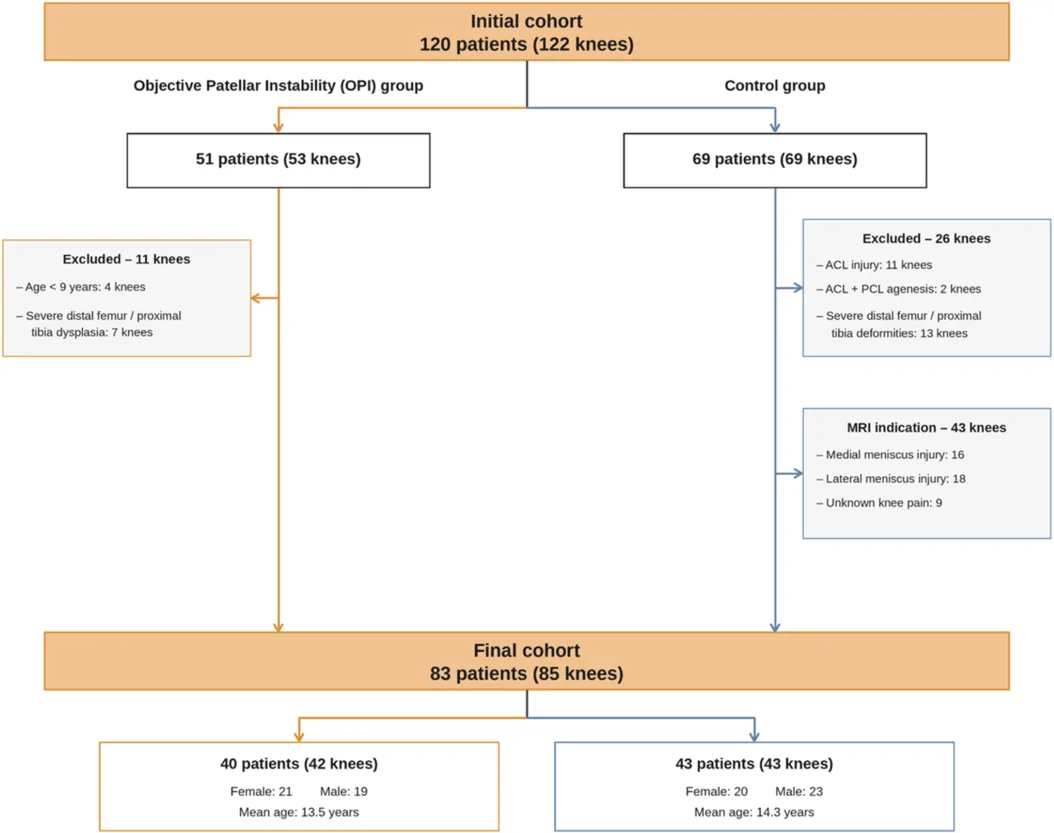
Flowchart illustrating patient selection and cohort formation. From an initial cohort of 120 patients (122 knees), 83 patients (85 knees) met the inclusion criteria and were included in the final analysis: 40 patients (42 knees) in the OPI group and 43 patients (43 knees) in the control group. ACL, anterior cruciate ligament; OPI, objective patellar instability; PCL, posterior cruciate ligament.

This retrospective comparative study included paediatric patients (<18 years) with both distal femoral and tibial physis open who underwent knee MRI between January 2021 and December 2025. Skeletal maturity was evaluated on MRI through assessment of the proximal tibial physis following the criteria established by Fabricant et al. based on the Pennock bone age atlas [26]. Physes were classified as open when a continuous low‐signal physeal line was visible throughout the entire growth plate. Loss of continuity of this signal, either partially or completely, was interpreted as physeal closure progression; therefore, partially fused physes and those showing only a residual physeal scar were grouped as closing/closed. The OPI group comprised patients with ≥2 documented lateral patellar dislocations (19 males, 21 females). Controls underwent MRI for non‐patellofemoral indications and had no history of instability or surgery (medial meniscal tear, *n *= 16; lateral meniscal tear, *n* = 18; non‐specific knee pain, *n* = 9; 20 males, 23 females). Exclusion criteria included prior surgery, major ligament injury (i.e., anterior cruciate ligament [ACL] lesions), inflammatory or neuromuscular disorders and advanced cartilage degeneration (i.e., ICRS > 2). Patients younger than 9 years were excluded because incomplete patellar ossification at this age prevents reliable identification of patellar landmarks and accurate MRI‐based measurements of patellar height and engagement indices (Figure 1). Baseline characteristics of both groups are reported in Table 1. In the entire cohort, all 83 patients had open physes on MRI (40 OPI and 43 controls). Two OPI patients were assessed bilaterally, yielding 85 knees (42 OPI and 43 controls) for analysis. Physeal status was assessed on MRI at the proximal tibia according to the consensus criteria of Fabricant et al., which are based on the Pennock bone‐age atlas [26]. Physes were classified as open when the low‐signal physeal band could be traced continuously along the entire proximal tibial physis on intermediate‐weighted (intermediate echo‐time) sequences, and as closing/closed when this band was no longer continuous (closing) or only a physeal scar remained (closed). Partially closed physes were assigned to the closing/closed group, in accordance with Fabricant et al.

**Table 1:** Baseline characteristics of the two groups.

Characteristic	OPI (42 knees)	Control (43 knees)	*p*
Patients, *n*	40 (2 bilateral)	43	—
Age, years	14.31 [13.00–16.05]	12.65 [11.26–14.05]	<0.001
Sex, male/female	19/21	20/23	—
Skeletal maturity	All physes open	All physes open	—
Trochlear shape, concave/flat/convex	19/16/7	42/1/0	—

*Note*: Age is reported as median [interquartile range] and compared with the Wilcoxon rank‐sum test (Hodges–Lehmann median difference 1.76 years, 95% CI 1.06–2.52). Sex is recorded at patient level. Control indications for MRI were medial meniscal tear (*n* = 16), lateral meniscal tear (*n* = 18) and non‐specific knee pain (*n* = 9).

Abbreviations: CI, confidence interval; OPI, objective patellar instability.

### MRI protocol

All MRIs were acquired with the knee in near‐full extension using a 1.5‐T scanner and a dedicated knee coil (MRI coil protocol with standardized lower limb and foot rotations). Sagittal fat‐suppressed proton density sequences were used without venous para‐magnetic contrast agents. All MRI examinations were performed using standardized protocols. Measurements were obtained on sagittal sequences using a cartilage‐based approach.

### Measurement protocol

**Figure 2:**
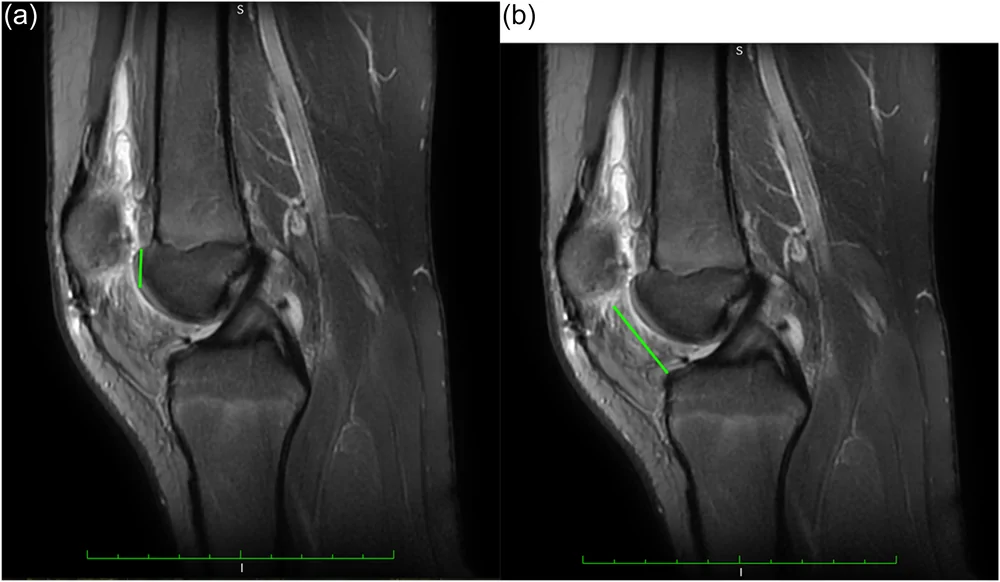
Sagittal trochlear cartilage length (STCL) (a) and patello‐tibial distance (PTD) (b) measured on an OPI‐right knee. OPI, objective patellar instability.

**Figure 3:**
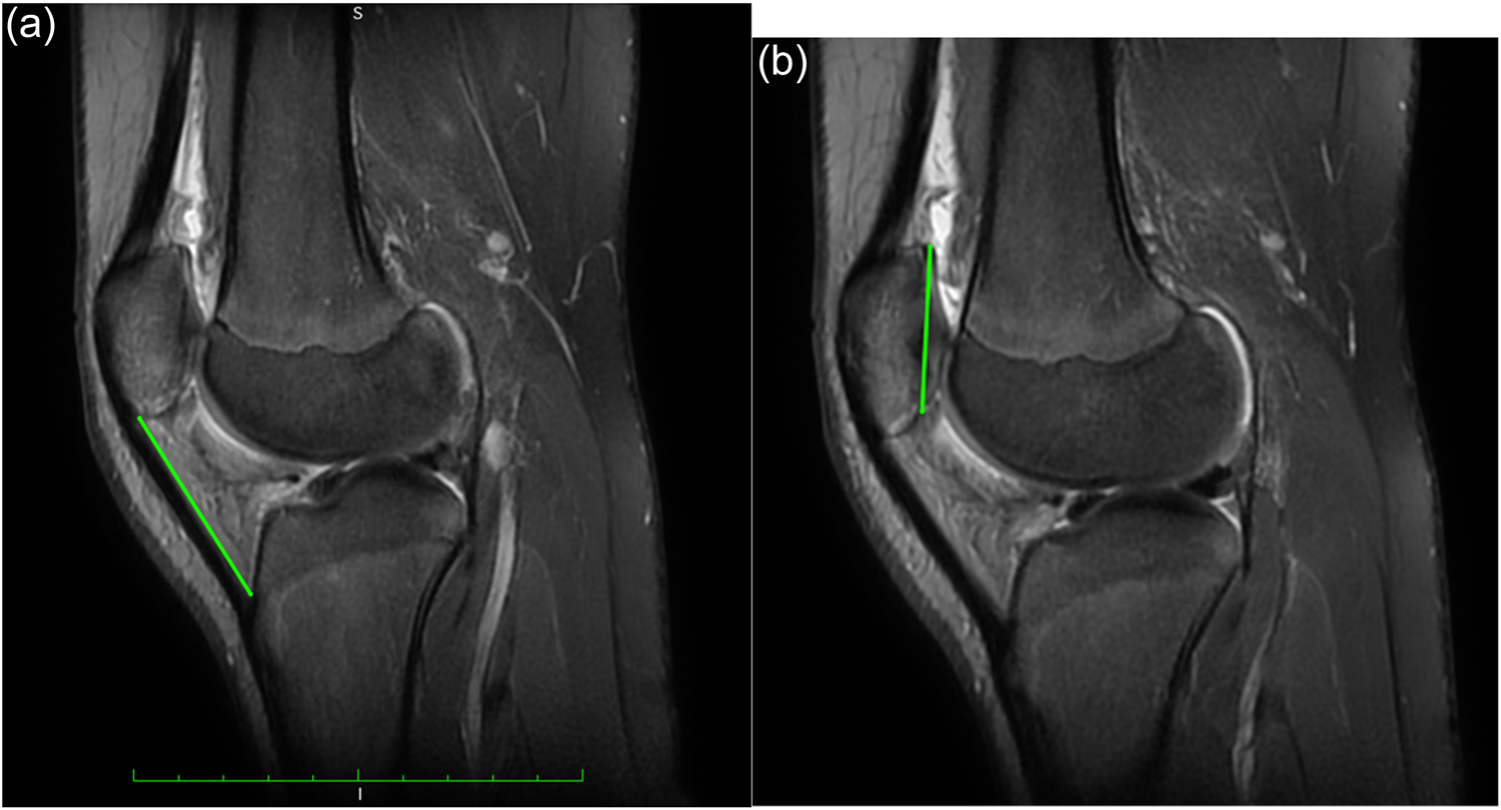
Patellar tendon length (PTL) (a) and sagittal patellar cartilage length (SPCL) (b) measured on an OPI‐right knee. OPI, objective patellar instability.

**Figure 4:**
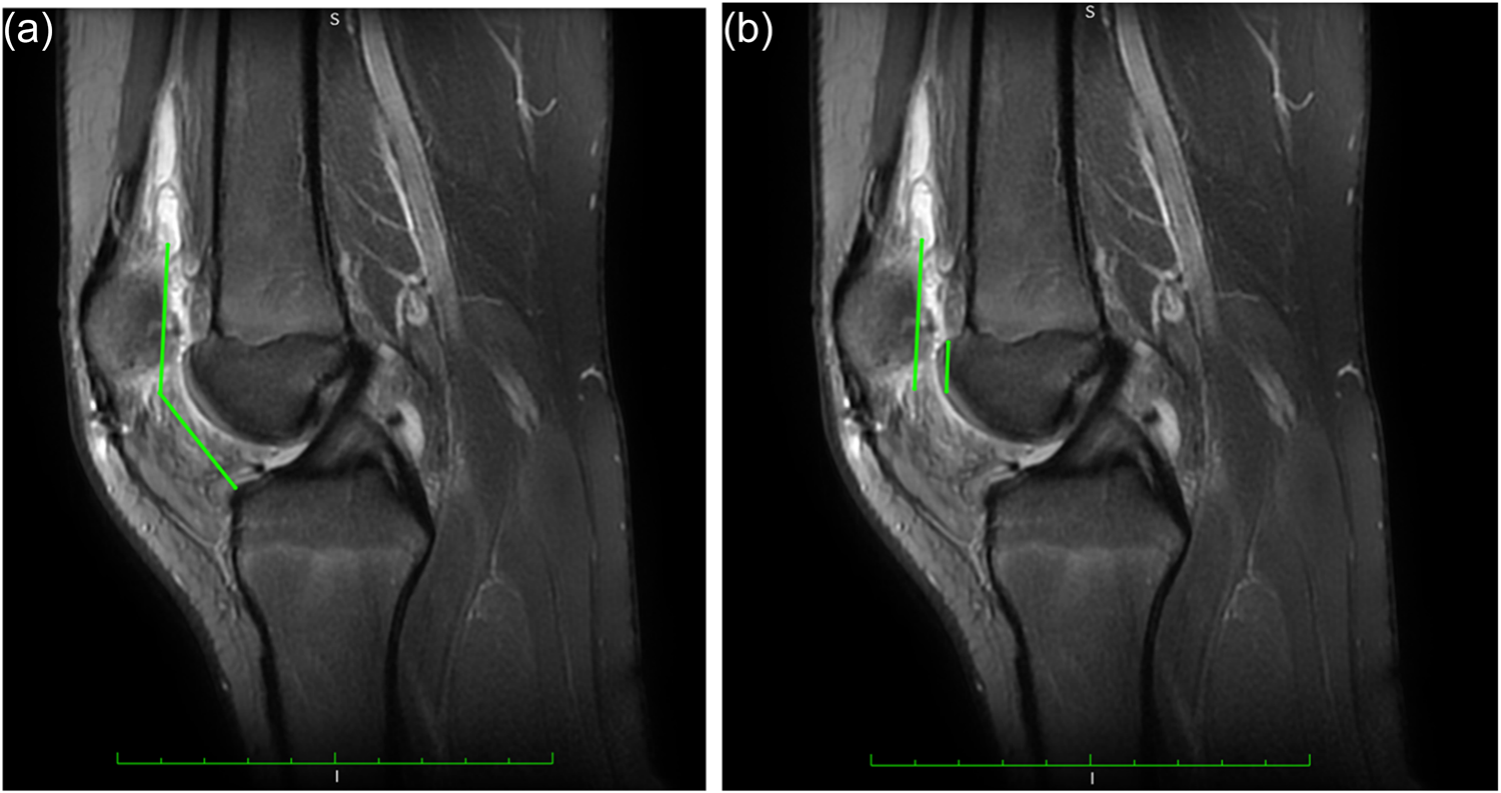
MRI‐sagittal patellar height index (sPHI) (a) and sagittal patellar engagement (SPE) (b) measured on an OPI‐right knee. OPI, objective patellar instability.

Three independent observers performed measurements following a standardized protocol. Radiological measurements were obtained using Horos software (Version 3.3.6; Horos Project) following the sagittal MRI protocol described by Dejour et al. [10, 12]. Patellar height index (PHI) and SPE were calculated using cartilage‐based landmarks on defined sagittal slices following the multi‐slice protocol (Figure 2). In particular, SPCL was measured on the slice where the patellar cartilage is the longest on the sagittal plane (sagittal patellar slice), whilst patello‐tibial distance (PTD) and sagittal trochlear cartilage length (STCL) were measured on the slice where the ACL is clearly visible from tibia to femur on the sagittal plane (center of the knee slice). Patellar tendon length (PTL) (Figure 3) was measured on standardized MRIs in which the tendon was taut. Absolute measures included SPCL (Figure 3), STCL and PTD (Figure 4). The three observers (a paediatric orthopaedic consultant, a knee surgery fellow and a musculoskeletal radiologist) were blinded to group allocation and to each other's measurements. For every knee and every parameter, the value entered into the analysis was the arithmetic mean of the three independent measurements; no consensus reading, senior‐author adjudication or re‐measurement of discordant cases was performed. A senior author reviewed the measurement protocol for methodological consistency but did not modify the recorded values. The complete observer‐level dataset, together with the final averaged value used for analysis, is provided as Data S1.

### Reliability and statistical analysis

Interobserver reliability was assessed using intraclass correlation coefficients (ICCs) calculated with a two‐way random‐effects model and an absolute‐agreement definition consistent with methodological recommendations and previous 147 reliability studies of orthopaedic and patellofemoral measurements [15, 19, 27, 31]. Both single‐measure ICC(A,1), reflecting the reliability of a single observer, and average‐measure ICC(A,k), reflecting the reliability of the three‐observer mean actually used in the analyses, are reported with their 95% confidence intervals (CIs). Reliability was classified according to Koo and Li as poor (<0.50), moderate (0.50–0.74), good (0.75–0.89) or excellent (≥0.90); the average‐measure coefficient was used for this classification. Continuous variables were described using means, standard deviations (SDs), ranges, medians and interquartile ranges (IQRs), whereas categorical variables were reported as frequencies and percentages. Between‐group comparisons of continuous variables were performed using Student's *t* test for normally distributed data and the Wilcoxon rank‐sum test for non‐normally distributed data. Normality was verified with the Shapiro–Wilk test. Effect sizes were expressed as mean differences (MDs) with corresponding 95% CIs. For variables compared with the rank‐sum test, the Hodges–Lehmann median difference and its 95% CI are reported.

Comparisons of categorical variables were conducted using the Kruskal–Wallis test, with effect sizes reported as odds ratios (ORs) and 95% CIs. Similarly, differences between knees classified as patella norma and those with functional patella alta were assessed using Student's *t* test or the Wilcoxon rank‐sum test, as appropriate, for continuous variables and the Kruskal–Wallis test for categorical variables. Effect sizes were reported as MDs or ORs, respectively, together with their 95% CIs.

Univariable and multivariable logistic regression analyses were performed to investigate the associations of patellar height parameters—including the PHI, SPE index and the presence of functional patella alta—as well as absolute patellar height measurements (patellar cartilage length, PTD, STCL and PTL) and trochlear morphology, with the occurrence of OPI. ORs for continuous predictors are expressed per one‐SD increase to allow comparison across parameters measured on different scales. To limit the risk of overfitting, candidate predictors were first screened univariably and the multivariable model was deliberately restricted to the two prespecified indices of interest, PHI and SPE; with 42 events this corresponds to 21 events per variable, well above the conventional minimum of ten. Collinearity between PHI and SPE was assessed before entering both terms in the same model, using the Pearson correlation coefficient and the variance inflation factor (VIF), with a predefined threshold for concern of |*r*| > 0.70 or VIF > 5. The interquartile reference thresholds derived from the control group—PHI of 1.17 (75th percentile) and SPE of 0.35 (25th percentile)—were used to define normal patellofemoral morphology, characterized by a PHI ≤ 1.17 and an SPE ≥ 0.35. Conversely, functional patella alta was defined by the simultaneous presence of a PHI > 1.17 and an SPE < 0.35. A *p* value < 0.05 was considered significant. The exact percentile values in the control group were 1.175 for PHI and 0.350 for SPE; these were rounded to 1.17 and 0.35, and the rounded values were applied consistently in all analyses involving functional patella alta. Non‐normally distributed variables are reported as medians with IQRs. Analyses were performed in Python 3.12 (pingouin 0.6 and statsmodels 0.14).

## RESULTS

### Patient selection and baseline characteristics

Eighty‐three patients met the inclusion criteria and were analysed, of whom two OPI patients were assessed bilaterally, giving 85 knees: 42 OPI knees (40 patients) and 43 control knees (43 patients). All physes were open on MRI in both groups. The mean age of the whole cohort was 13.44 ± 1.90 years (range 9.3–16.7). OPI knees belonged to older patients than control knees (median 14.31 years, IQR 13.00–16.05, vs. 12.65 years, IQR 11.26–14.05; Hodges–Lehmann median difference 1.76 years, 95% CI 1.06–2.52; *p* < 0.001), so the two groups were not matched for age. Sex distribution was comparable (OPI 19 males/21 females; controls 20 males/23 females). Trochlear morphology differed markedly between groups: A concave trochlea was observed in 42 of 43 control knees, whereas OPI knees showed a concave trochlea in 19, a flat trochlea in 16 and a convex trochlea in seven cases. Baseline characteristics and between‐group comparisons are detailed in Table 1.

### Sagittal patellar height and engagement

OPI knees demonstrated a higher PHI than control knees (1.178 ± 0.171 vs. 1.083 ± 0.132; MD 0.095, 95% CI 0.029–0.161; *p* = 0.005) and a lower SPE (0.354 ± 0.207 vs. 0.484 ± 0.200; MD −0.130, 95% CI −0.218 to −0.042; *p* = 0.004). Among the absolute measurements, PTD (35.82 ± 5.66 vs. 30.74 ± 3.39 mm; *p* < 0.001), PTL (51.70 ± 8.36 vs. 43.30 ± 5.48 mm; *p* < 0.001) and SPCL (median 30.84 mm, IQR 28.76–32.55, vs. 28.35 mm, IQR 27.08–30.11; *p* = 0.002) were greater in OPI knees, whereas STCL was shorter (10.77 ± 6.24 vs. 13.75 ± 5.60 mm; *p* = 0.023). OPI knees also showed a greater TT–TG distance, a greater sulcus angle, a greater lateral patellar tilt and a lower lateral trochlear inclination (all *p* < 0.001). All between‐group comparisons are reported in Table 2.

**Table 2:** Sagittal MRI measurements in OPI and control knees.

Parameter	OPI (42 knees)	Control (43 knees)	Difference	95% CI	*p*
PHI (PTD/SPCL)	1.178 ± 0.171	1.083 ± 0.132	0.095	0.029 to 0.161	0.005
SPE (STCL/SPCL)	0.354 ± 0.207	0.484 ± 0.200	−0.130	−0.218 to −0.042	0.004
SPCL (mm)	30.84 [28.76–32.55]	28.35 [27.08–30.11]	2.14	0.85 to 3.33	0.002
PTD (mm)	35.82 ± 5.66	30.74 ± 3.39	5.08	3.06 to 7.10	<0.001
STCL (mm)	10.77 ± 6.24	13.75 ± 5.60	−2.98	−5.54 to −0.42	0.023
PTL (mm)	51.70 ± 8.36	43.30 ± 5.48	8.40	5.33 to 11.46	<0.001
CPs (mm)	5.51 ± 1.92	3.27 ± 1.38	2.24	1.52 to 2.96	<0.001
TT–TG (mm)	13.21 ± 4.16	8.08 ± 3.40	5.13	3.49 to 6.77	<0.001
LTI (°)	12.06 ± 5.42	18.42 ± 3.65	−6.36	−8.37 to −4.34	<0.001
Sulcus angle (°)	158.05 [152.30–162.50]	145.56 [142.02–148.98]	11.80	8.87 to 14.94	<0.001
Lateral patellar tilt (°)	18.63 ± 8.51	10.56 ± 3.81	8.08	5.22 to 10.93	<0.001

*Note*: Values are mean ± standard deviation, or median [interquartile range] for variables that did not satisfy the Shapiro–Wilk normality assumption (SPCL and sulcus angle), which were compared with the Wilcoxon rank‐sum test; all other comparisons used Student's *t* test. The difference column reports the mean difference (OPI − control) for *t* tested variables and the Hodges–Lehmann median difference for rank‐sum‐tested variables. Values are the mean of three independent observers.

Abbreviations: CI, confidence interval; CPs, cartilage patellar step; LTI, lateral trochlear inclination; MRI, magnetic resonance imaging; OPI, objective patellar instability; PHI, patellar height index; PTD, patello‐tibial distance; PTL, patellar tendon length; SPCL, sagittal patellar cartilage length; SPE, sagittal patellofemoral engagement; STCL, sagittal trochlear cartilage length; TT–TG, tibial tubercle–trochlear groove.

### Functional patella alta

Applying the control‐derived thresholds, functional patella alta (PHI > 1.17 and SPE < 0.35) was identified in 16 of 85 knees (18.8%), comprising 14 of 42 OPI knees (33.3%) and 2 of 43 control knees (4.7%). Knees with functional patella alta showed greater PTD (median 38.86 mm, IQR 37.05–41.58, vs. 31.00 mm, IQR 29.54–33.85; *p* < 0.001), greater PTL (56.76 ± 7.17 vs. 45.32 ± 6.82 mm; *p* < 0.001), shorter STCL (6.65 ± 2.91 vs. 13.58 ± 5.88 mm; *p* < 0.001) and a greater TT–TG distance (14.54 ± 4.73 vs. 9.70 ± 4.05 mm; *p* < 0.001), whereas SPCL did not differ (median 29.67 mm, IQR 28.90–31.49, vs. 29.31 mm, IQR 27.29–31.77; *p* = 0.415). As PHI and SPE define the two groups, their differences are reported in Table 3 for completeness only and are not interpretable as independent findings.

**Table 3:** Comparison between knees with functional patella alta and knees with patella norma.

Parameter	Functional patella alta (16 knees)	Patella norma (69 knees)	*p*
PHI (PTD/SPCL)	1.332 ± 0.131	1.083 ± 0.125	<0.001
SPE (STCL/SPCL)	0.220 ± 0.091	0.466 ± 0.206	<0.001
PTD (mm)	38.86 [37.05–41.58]	31.00 [29.54–33.85]	<0.001
PTL (mm)	56.76 ± 7.17	45.32 ± 6.82	<0.001
STCL (mm)	6.65 ± 2.91	13.58 ± 5.88	<0.001
SPCL (mm)	29.67 [28.90–31.49]	29.31 [27.29–31.77]	0.415
TT–TG (mm)	14.54 ± 4.73	9.70 ± 4.05	<0.001
Lateral patellar tilt (°)	20.10 [14.08–25.50]	12.34 [9.67–16.22]	0.003
LTI (°)	12.36 ± 4.87	16.01 ± 5.54	0.018
Sulcus angle (°)	155.26 [152.10–160.36]	148.74 [143.76–155.91]	0.059

*Note*: Functional patella alta was defined as PHI > 1.17 and SPE < 0.35. Values are mean ± standard deviation or median [interquartile range] for non‐normally distributed variables. Because PHI and SPE constitute the classification criteria, the differences observed for these two indices follow by construction and are reported for completeness only; they are not independent findings. Functional patella alta was present in 14 of 42 OPI knees (33.3%) and in 2 of 43 control knees (4.7%).

Abbreviations: LTI, lateral trochlear inclination; OPI, objective patellar instability; PHI, patellar height index; PTD, patello‐tibial distance; PTL, patellar tendon length; SPCL, sagittal patellar cartilage length; SPE, sagittal patellofemoral engagement; STCL, sagittal trochlear cartilage length; TT–TG, tibial tubercle–trochlear groove.

### Interobserver reliability

Interobserver reliability of the three‐observer mean (average‐measure ICC) was excellent for SPE (0.953, 95% CI 0.93–0.97), STCL (0.954, 95% CI 0.93–0.97), PTD (0.944, 95% CI 0.90–0.97), PTL (0.968, 95% CI 0.95–0.98) and TT–TG distance (0.919, 95% CI 0.88–0.95); good for SPCL (0.797, 95% CI 0.70–0.86) and lateral trochlear inclination (0.773, 95% CI 0.66–0.85); and moderate for PHI (0.715, 95% CI 0.58–0.81), sulcus angle (0.665, 95% CI 0.50–0.78) and lateral patellar tilt (0.714, 95% CI 0.58–0.81). Single‐measure coefficients were correspondingly lower, and were 0.455 (95% CI 0.32–0.59) for PHI and 0.872 (95% CI 0.82–0.91) for SPE. Complete ICC values, with model, agreement type and both single‐ and average‐measure specifications, are reported in Table 4; observer‐level data are provided as Data S1.

**Table 4:** Interobserver reliability of the MRI measurements.

Parameter	Knees, *n*	ICC single measure (95% CI)	ICC average measure (95% CI)	Agreement
PHI (PTD/SPCL)	81	0.455 (0.32–0.59)	0.715 (0.58–0.81)	Moderate
SPE (STCL/SPCL)	81	0.872 (0.82–0.91)	0.953 (0.93–0.97)	Excellent
SPCL (mm)	81	0.567 (0.44–0.68)	0.797 (0.70–0.86)	Good
PTD (mm)	81	0.848 (0.76–0.90)	0.944 (0.90–0.97)	Excellent
STCL (mm)	81	0.875 (0.82–0.91)	0.954 (0.93–0.97)	Excellent
PTL (mm)	73	0.911 (0.87–0.94)	0.968 (0.95–0.98)	Excellent
CPs (mm)	78	0.914 (0.87–0.95)	0.970 (0.95–0.98)	Excellent
TT–TG (mm)	81	0.792 (0.72–0.85)	0.919 (0.88–0.95)	Excellent
LTI (°)	75	0.531 (0.40–0.65)	0.773 (0.66–0.85)	Good
Sulcus angle (°)	71	0.398 (0.25–0.54)	0.665 (0.50–0.78)	Moderate
Lateral patellar tilt (°)	81	0.454 (0.32–0.58)	0.714 (0.58–0.81)	Moderate

*Note*: ICCs were calculated with a two‐way random‐effects model and an absolute‐agreement definition, using the measurements of three independent observers. The single‐measure coefficient ICC(A,1) expresses the reliability of one observer; the average‐measure coefficient ICC(A,k) expresses the reliability of the three‐observer mean used in all analyses and was used to classify agreement according to Koo and Li as poor (<0.50), moderate (0.50–0.74), good (0.75–0.89) or excellent (≥0.90). Four control knees for which the third observer did not perform independent measurements were excluded from the ICC computation, which was restricted to complete observer triplets.

Abbreviations: CI, confidence interval; CPs, cartilage patellar step; ICC, intraclass correlation coefficient; LTI, lateral trochlear inclination; MRI, magnetic resonance imaging; PHI, patellar height index; PTD, patello‐tibial distance; PTL, patellar tendon length; SPCL, sagittal patellar cartilage length; SPE, sagittal patellofemoral engagement; STCL, sagittal trochlear cartilage length; TT–TG, tibial tubercle–trochlear groove.

### Logistic regression

In univariable analysis, higher PHI (OR 1.95 per SD, 95% CI 1.19–3.21; *p* = 0.008) and lower SPE (OR 0.51 per SD, 95% CI 0.31–0.83; *p* = 0.006) were associated with OPI, as were PTD (OR 3.89, 95% CI 1.97–7.67), PTL (OR 4.19, 95% CI 2.11–8.33) and TT–TG distance (OR 4.79, 95% CI 2.41–9.53; all *p* < 0.001). Functional patella alta was associated with instability with an OR of 10.25 (95% CI 2.16–48.66; *p* = 0.003). PHI and SPE were only weakly correlated (Pearson *r* = −0.264; *p* = 0.015) and showed no collinearity (VIF 1.07), permitting their simultaneous inclusion. In the multivariable model, both indices remained independently associated with OPI (PHI: OR 1.78 per SD, 95% CI 1.05–3.01, *p* = 0.032; SPE: OR 0.57 per SD, 95% CI 0.34–0.93, *p* = 0.025). In a sensitivity analysis additionally adjusted for age, the associations of PHI and SPE were attenuated and no longer reached statistical significance, whereas PTD, PTL and TT–TG distance remained independently associated with instability (Table S1). Full regression results are presented in Table 5.

**Table 5:** Logistic regression for objective patellar instability.

Predictor	Odds ratio per 1 SD (95% CI)	*p*
(a) Univariable analysis		
PHI (PTD/SPCL)	1.95 (1.19–3.21)	0.008
SPE (STCL/SPCL)	0.51 (0.31–0.83)	0.006
SPCL (mm)	1.89 (1.16–3.08)	0.010
PTD (mm)	3.89 (1.97–7.67)	<0.001
STCL (mm)	0.59 (0.37–0.94)	0.027
PTL (mm)	4.19 (2.11–8.33)	<0.001
TT–TG (mm)	4.79 (2.41–9.53)	<0.001
LTI (°)	0.18 (0.08–0.39)	<0.001
Sulcus angle (°)	7.97 (3.23–19.66)	<0.001
Lateral patellar tilt (°)	5.98 (2.52–14.18)	<0.001
(b) Multivariable analysis		
PHI (PTD/SPCL)	1.78 (1.05–3.01)	0.032
SPE (STCL/SPCL)	0.57 (0.34–0.93)	0.025

*Note*: Odds ratios for continuous predictors are expressed per one standard deviation increase. Multivariable model: *n* = 85, events = 42, EPV = 21, corresponding to 21 events per variable. Collinearity between PHI and SPE was excluded before fitting the model (Pearson *r* = −0.264 (*p* = 0.015); VIF = 1.07). Functional patella alta, entered as a binary predictor, was associated with instability with an odds ratio of 10.25 (95% CI 2.16–48.66), *p* = 0.003.

Abbreviations: CI, confidence interval; EPV, events per variable; LTI, lateral trochlear inclination; PHI, patellar height index; PTD, patello‐tibial distance; PTL, patellar tendon length; SD, standard deviation; SPCL, sagittal patellar cartilage length; SPE, sagittal patellofemoral engagement; STCL, sagittal trochlear cartilage length; TT–TG, tibial tubercle–trochlear groove; VIF, variance inflation factor.

## DISCUSSION

The principal finding of this study is that paediatric recurrent patellar instability is associated with increased sagittal patellar height and reduced SPE on MRI. This is consistent with previous evidence identifying patella alta, trochlear dysplasia, TT–TG distance and other MRI‐defined morphological abnormalities as relevant features of paediatric patellofemoral instability [2, 6, 9, 16, 29]. Knees with OPI showed higher PHI values and lower SPE values than control knees, indicating that altered sagittal patellofemoral relationships accompany instability in skeletally immature patients (Table 2). Exploratory thresholds of PHI > 1.17 and SPE < 0.35, derived from the interquartile limits of the present control group, identified a subgroup of knees with functional patella alta that was over‐represented among unstable knees.

As shown in Table 3, knees classified as having functional patella alta differed from knees with patella norma in several sagittal parameters that are independent of the defining criteria: PTD and PTL were greater and STCL was shorter, while the TT–TG distance was also increased. Because functional patella alta is defined by PHI and SPE, the higher PHI and lower SPE observed in this subgroup, by construction, are therefore not presented as independent findings. In contrast, SPCL did not differ significantly between the two subgroups, suggesting that patellar cartilage length itself contributes little to the sagittal configuration associated with instability.

Patellar height has long been recognized as a key anatomical risk factor for patellar instability [9, 30, 35]. Classical radiographic indices such as the Insall–Salvati and Blackburne–Peel ratios have historically been used to quantify patellar height [5, 17, 25, 28]. However, these tibial‐referenced measurements depend on landmarks and proportions that evolve during skeletal growth [29, 34], and values obtained on radiographs and on MRI are not necessarily interchangeable [20, 23, 36]. Both considerations complicate their interpretation in skeletally immature patients.

MRI provides a comprehensive assessment of patellofemoral morphology and alignment while avoiding ionizing radiation, which is particularly relevant in paediatric populations [18, 21]. The concept of SPE, introduced by Dejour and colleagues, integrates patellar height relative to both tibial and trochlear landmarks and allows identification of functional patella alta [10, 12]. The present results suggest that this MRI‐based framework can also be applied to skeletally immature patients. This is consistent with the wider use of MRI to characterise developmental trochlear and cartilage morphology [1, 3, 14, 32, 34], quantitative trochlear dysplasia [6, 13, 16, 33] and soft‐tissue abnormalities associated with paediatric patellofemoral instability [7].

Interobserver reliability differed appreciably between parameters. The engagement‐related measurements were the most reproducible: SPE, STCL, PTD and PTL all reached excellent average‐measure agreement. By contrast, PHI showed only moderate agreement, reflecting the fact that it combines two independently identified landmarks and is therefore exposed to the variability of both. Previous studies have likewise shown that the reliability of patellar height and other radiological measurements can vary substantially according to the method, imaging modality and observer [15, 19, 27, 31]. This observation tempers the interpretation of PHI as a stand‐alone diagnostic index in the paediatric knee and supports the practice of averaging measurements from more than one observer or of relying preferentially on the more reproducible engagement parameters, when these indices are used clinically.

From a clinical perspective, the thresholds derived here should be regarded as exploratory, cohort‐derived values rather than established reference values and require external validation before being applied to other populations. In particular, the combination of increased patellar height and reduced sagittal engagement may indicate insufficient trochlear engagement during early knee flexion. Therefore, these MRI‐based parameters may help guide risk stratification and surgical decision‐making, particularly when evaluating the need for procedures aimed at correcting patellar height or improving patellofemoral engagement within the broader principles described for paediatric and adolescent patellar instability management [8, 11, 30, 35]. In skeletally immature knees, such decisions remain constrained by the open physes, and soft‐tissue reconstruction continues to be a central surgical option in this age group [24].

However, further prospective studies are required to determine whether these thresholds can be reliably used to define operative indications in paediatric patellar instability.

Instability in the present paediatric cohort occurred at lower sagittal engagement values than those reported in adults. In the adult cross‐sectional study of Dejour et al., which applied the same standardised MRI protocol, unstable knees showed a PHI of 1.18 ± 0.21 and an SPE of 0.42 ± 0.11; these are descriptive group values for an adult OPI population and not diagnostic thresholds [12]. A previous consensus description of the Lyon protocol proposed that an SPE below 0.45 in a patient with patellar dislocation may indicate patella alta with insufficient functional sagittal engagement [11]. The corresponding values in the present paediatric cohort (PHI 1.178 ± 0.171; SPE 0.354 ± 0.207) suggest that age‐related anatomical and biomechanical factors influence patellofemoral stability. Developmental changes in trochlear morphology during growth may partly explain these findings, as paediatric imaging studies have demonstrated age‐dependent evolution of trochlear anatomy and patellar instability risk factors [1, 3, 14, 29, 34]. Additionally, soft‐tissue stabilizers such as the medial patellofemoral complex may play an important role in maintaining patellar stability in younger patients, consistent with MRI findings involving the lateral retinaculum and favourable outcomes reported for physeal‐sparing MPFL reconstruction [7, 24].

From a clinical perspective, these findings highlight the importance of incorporating MRI‐based sagittal engagement parameters into the evaluation of paediatric patellar instability, rather than relying solely on patellar height measurements. Assessing patellofemoral engagement may therefore improve risk stratification and surgical decision‐making in this population.

## LIMITATIONS

Limitations include the retrospective design, potential slice‐selection variability, lack of sex‐specific analyses, homogeneous ethnicity and modest sample size; population‐related morphological differences should also be considered when interpreting knee measurements across cohorts [22]. The OPI and control groups were not matched for age, and control patients were, on average, younger; since patellar height and trochlear morphology both evolve during skeletal growth, age may act as a confounder for the observed associations, and the differences reported here should be interpreted with this in mind. Reliability was not uniform across parameters, and the moderate agreement observed for PHI indicates that this index should not be used in isolation in keeping with previous reports of variable interobserver reliability for patellofemoral measurements [15, 19, 27, 31]. The thresholds proposed here were derived from the distribution of the present control group and are therefore exploratory; trochlear dysplasia was characterised by trochlear shape and by quantitative angular parameters rather than by the Dejour grade and published normal thresholds have been shown to vary with measurement technique, landmarks and sex [33]. Given these considerations, thresholds should be further validated in a homogeneous and larger cohort. Despite these limitations, the use of a standardised, blinded, three‐observer measurement protocol supports the internal consistency of the findings.

## CONCLUSION

**Figure 5:**
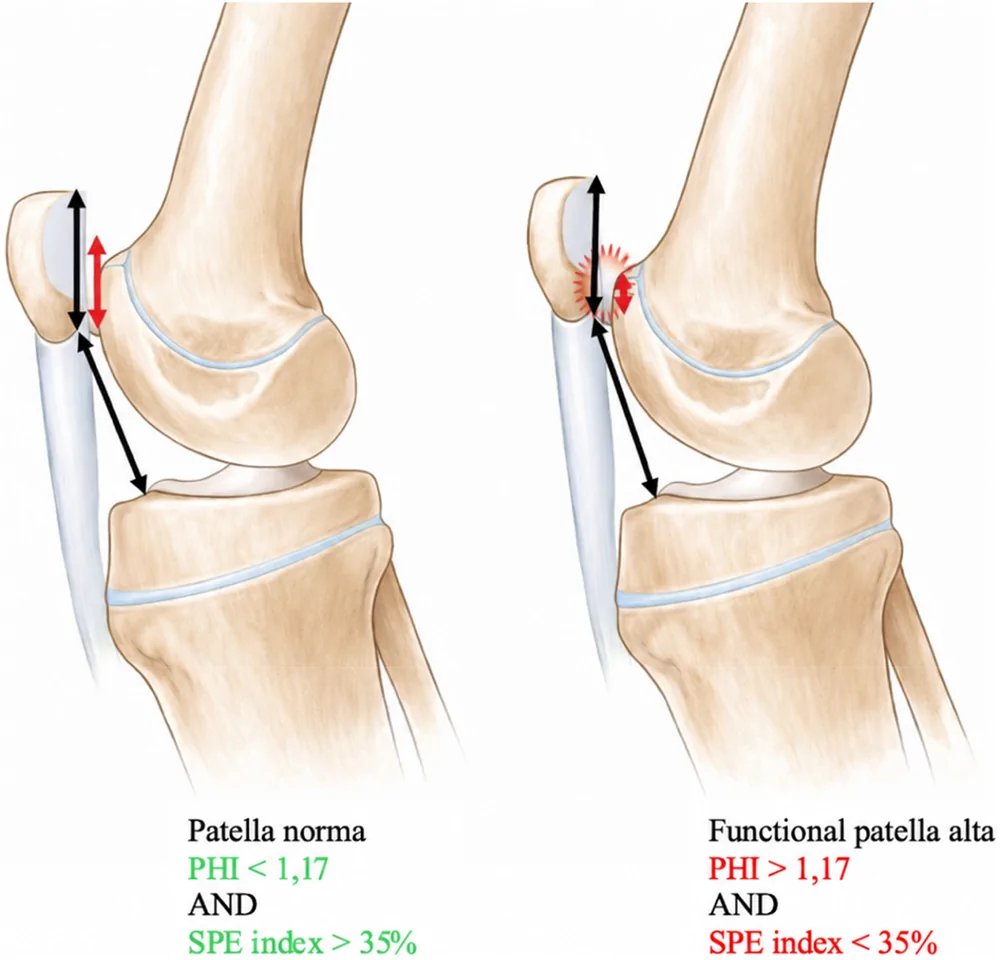
Differences in PHI index and SPE index between a skeletally‐immature patella norma and skeletally‐immature functional patella alta. PHI, patellar height index; SPE, sagittal patellofemoral engagement.

Paediatric recurrent patellar dislocation is characterized by increased patellar height and reduced sagittal trochlear engagement on MRI. Although functional patella alta is applicable in skeletally immature knees, paediatric instability occurs at lower engagement values than those described in adults. The cohort‐derived thresholds proposed here (PHI > 1.17 and SPE < 0.35) should be regarded as exploratory and require external validation before paediatric‐specific MRI reference values can be established (Figure 5).

## AUTHOR CONTRIBUTIONS

**Francesco Puglia**: Conceptualization; investigation; methodology; validation; writing—review and editing. **Paolo Ferrua**: Conceptualization; methodology; validation; writing—review and editing. **David H. Dejour**: Revised the work. **Marco Nicoletti**: Investigation; methodology; writing—review and editing; writing—original draft. **Alessio Volpe**: Investigation; methodology; writing—review and editing; writing—original draft. **Riccardo Compagnoni**: Investigation; methodology; validation. **Antonio Memeo**: Investigation; resources; validation. **Pietro Simone Randelli**: Revised the work. All authors read and approved the final manuscript.

## FUNDING INFORMATION

The authors have no funding to report.

## CONFLICT OF INTEREST STATEMENT

The authors declare no conflicts of interest.

## ETHICS STATEMENT

All patients provided written informed consent to use their data and images for research and publishing purposes.

## Supporting information

Supporting File 1:

Supporting File 2:

Supporting File 3:

## Data Availability

The data that support the findings of this study are available from the corresponding author upon reasonable request.
