# Supplementary material for: Increased sagittal patellar height and reduced trochlear engagement on MRI are associated with recurrent patellar instability in skeletally immature knees: A comparative study
Source: J Exp Orthop. 2026 Aug 21;13(3):e70896. doi: 10.1002/jeo2.70896 (PMC13495771; doi:10.1002/jeo2.70896)
Supplement: Supplementary file 1 [file JEO2-13-e70896-s002.docx]

**Statistical Results Package — Revised Analysis**

*Sagittal MRI-Based Patellar Height and Trochlear Engagement in Skeletally Immature Knees With Recurrent Patellar Instability*

Analysis re-run on the corrected database (MISURE_COMPLETE_FEMORO_ROTULEA_PED_2.xlsx) — 85 knees (42 OPI / 43 control), 3 independent observers

**Analytical notes (for Methods)**

• Final value per knee and per parameter = arithmetic mean of the three independent observers' measurements (MN, FP, AV). No consensus or senior-author adjudication was applied to the numeric values used for analysis.

• Interobserver reliability: two-way random-effects intraclass correlation coefficient (ICC), absolute-agreement definition. Single-measure ICC(A,1) reflects the reliability of one observer; average-measure ICC(A,k) reflects the reliability of the 3-observer mean actually used in the analysis. Interpretation (Koo & Li 2016): <0.50 poor, 0.50–0.74 moderate, 0.75–0.89 good, ≥0.90 excellent.

• For 4 control knees the third observer (AV) did not perform independent measurements; the two available observers were used for those knees, and they were excluded from the 3-observer ICC (ICC computed on complete triplets).

• Continuous variables: Student t-test if both groups passed Shapiro–Wilk normality, otherwise Wilcoxon rank-sum; effect size = mean difference (95% CI), Hodges–Lehmann median difference for non-normal variables. Logistic-regression odds ratios for continuous predictors are expressed per 1 SD increase.

**Table 1. Baseline characteristics by group**

| **Characteristic** | **OPI** | **Control** |
| --- | --- | --- |
| Knees, n | 42 | 43 |
| Patients, n | 40 (2 bilateral) | 43 |
| Age, years (median [IQR]) | 14.31 [13.00–16.05] | 12.65 [11.26–14.05] |
| Sex (M / F)* | 19 / 21 | 20 / 23 |
| Skeletal maturity | All physes open | All physes open |
| MRI indication | Recurrent patellar instability (≥2 dislocations) | Medial meniscus 16; lateral meniscus 18; non-specific knee pain 9 |
| Trochlear shape (concave / flat / convex) | 19 / 16 / 7 | 42 / 1 / 0 |

*Age difference between groups: Hodges–Lehmann median difference 1.76 years (95% CI 1.06 to 2.52), p<0.001 — the groups are NOT age-matched (OPI significantly older). *Sex recorded at patient level (from clinical records; not in the imaging dataset).*

**Table 2. MRI sagittal measurements — OPI vs Control (3-observer mean)**

| **Parameter** | **OPI (mean ± SD)** | **Control (mean ± SD)** | **MD** | **95% CI** | **p** |
| --- | --- | --- | --- | --- | --- |
| PHI (PTD/SPCL) | 1.178 ± 0.171 | 1.083 ± 0.132 | 0.095 | 0.029 to 0.161 | 0.005 |
| SPE (STCL/SPCL) | 0.354 ± 0.207 | 0.484 ± 0.200 | -0.130 | -0.218 to -0.042 | 0.004 |
| SPCL (mm) | 30.84 [28.76–32.55] | 28.35 [27.08–30.11] | 2.14 | 0.85 to 3.33 | 0.002 |
| PTD (mm) | 35.82 ± 5.66 | 30.74 ± 3.39 | 5.08 | 3.06 to 7.10 | <0.001 |
| STCL (mm) | 10.77 ± 6.24 | 13.75 ± 5.60 | -2.98 | -5.54 to -0.42 | 0.023 |
| PTL (mm) | 51.70 ± 8.36 | 43.30 ± 5.48 | 8.40 | 5.33 to 11.46 | <0.001 |
| CPs (mm) | 5.51 ± 1.92 | 3.27 ± 1.38 | 2.24 | 1.52 to 2.96 | <0.001 |
| TT–TG (mm) | 13.21 ± 4.16 | 8.08 ± 3.40 | 5.13 | 3.49 to 6.77 | <0.001 |
| LTI (°) | 12.06 ± 5.42 | 18.42 ± 3.65 | -6.36 | -8.37 to -4.34 | <0.001 |
| Sulcus angle (°) | 158.05 [152.30–162.50] | 145.56 [142.02–148.98] | 11.80 | 8.87 to 14.94 | <0.001 |
| Lateral patellar tilt (°) | 18.63 ± 8.51 | 10.56 ± 3.81 | 8.08 | 5.22 to 10.93 | <0.001 |

*MD = mean difference (OPI − control) for t-tested variables, Hodges–Lehmann median difference for rank-sum-tested variables (SPCL). All comparisons unadjusted for age.*

**Table 3. Functional patella alta vs patella norma**

| **Parameter** | **Functional patella alta (mean ± SD)** | **Patella norma (mean ± SD)** | **p** |
| --- | --- | --- | --- |
| PHI (PTD/SPCL) | 1.332 ± 0.131 | 1.083 ± 0.125 | <0.001 |
| SPE (STCL/SPCL) | 0.220 ± 0.091 | 0.466 ± 0.206 | <0.001 |
| PTD (mm) | 38.86 [37.05–41.58] | 31.00 [29.54–33.85] | <0.001 |
| PTL (mm) | 56.76 ± 7.17 | 45.32 ± 6.82 | <0.001 |
| STCL (mm) | 6.65 ± 2.91 | 13.58 ± 5.88 | <0.001 |
| SPCL (mm) | 29.67 [28.90–31.49] | 29.31 [27.29–31.77] | 0.415 |
| TT–TG (mm) | 14.54 ± 4.73 | 9.70 ± 4.05 | <0.001 |
| Lateral patellar tilt (°) | 20.10 [14.08–25.50] | 12.34 [9.67–16.22] | 0.003 |
| LTI (°) | 12.36 ± 4.87 | 16.01 ± 5.54 | 0.018 |
| Sulcus angle (°) | 155.26 [152.10–160.36] | 148.74 [143.76–155.91] | 0.059 |

*Functional patella alta defined as PHI > 1.17 AND SPE < 0.35 (control-group 75th/25th percentiles). Because PHI and SPE define the groups, their between-group differences are expected by definition and are reported for completeness only, not as independent findings. Functional patella alta: 14/42 OPI vs 2/43 control knees (16/85 overall, 18.8%). Thresholds applied as stated (1.17 / 0.35); exact control percentiles were 1.1746 and 0.3502.*

**Table 4. Interobserver reliability (ICC, two-way random, absolute agreement)**

| **Parameter** | **n knees** | **ICC single (95% CI)** | **ICC average (95% CI)** | **Agreement*** |
| --- | --- | --- | --- | --- |
| PHI (PTD/SPCL) | 81 | 0.455 (0.32–0.59) | 0.715 (0.58–0.81) | moderate |
| SPE (STCL/SPCL) | 81 | 0.872 (0.82–0.91) | 0.953 (0.93–0.97) | excellent |
| SPCL (mm) | 81 | 0.567 (0.44–0.68) | 0.797 (0.70–0.86) | good |
| PTD (mm) | 81 | 0.848 (0.76–0.90) | 0.944 (0.90–0.97) | excellent |
| STCL (mm) | 81 | 0.875 (0.82–0.91) | 0.954 (0.93–0.97) | excellent |
| PTL (mm) | 73 | 0.911 (0.87–0.94) | 0.968 (0.95–0.98) | excellent |
| CPs (mm) | 78 | 0.914 (0.87–0.95) | 0.970 (0.95–0.98) | excellent |
| TT–TG (mm) | 81 | 0.792 (0.72–0.85) | 0.919 (0.88–0.95) | excellent |
| LTI (°) | 75 | 0.531 (0.40–0.65) | 0.773 (0.66–0.85) | good |
| Sulcus angle (°) | 71 | 0.398 (0.25–0.54) | 0.665 (0.50–0.78) | moderate |
| Lateral patellar tilt (°) | 81 | 0.454 (0.32–0.58) | 0.714 (0.58–0.81) | moderate |

**Agreement classified on the average-measure ICC (reliability of the 3-observer mean used in analysis). Note: PHI shows only moderate reliability (single-measure 0.455; average-measure 0.715) — this does NOT support the manuscript's current claim of 'excellent' PHI reliability and must be corrected. SPE, PTD, STCL and PTL are excellent; SPCL is good.*

**Table 5. Logistic regression for objective patellar instability**

**5a. Univariable (odds ratio per 1 SD increase)**

| **Predictor** | **OR (95% CI) per SD** | **p** |
| --- | --- | --- |
| PHI (PTD/SPCL) | 1.95 (1.19–3.21) | 0.008 |
| SPE (STCL/SPCL) | 0.51 (0.31–0.83) | 0.006 |
| SPCL (mm) | 1.89 (1.16–3.08) | 0.010 |
| PTD (mm) | 3.89 (1.97–7.67) | <0.001 |
| STCL (mm) | 0.59 (0.37–0.94) | 0.027 |
| PTL (mm) | 4.19 (2.11–8.33) | <0.001 |
| TT–TG (mm) | 4.79 (2.41–9.53) | <0.001 |
| LTI (°) | 0.18 (0.08–0.39) | <0.001 |
| Sulcus angle (°) | 7.97 (3.23–19.66) | <0.001 |
| Lateral patellar tilt (°) | 5.98 (2.52–14.18) | <0.001 |

**5b. Multivariable model: PHI + SPE (per 1 SD)**

| **Predictor** | **OR (95% CI) per SD** | **p** |
| --- | --- | --- |
| PHI (PTD/SPCL) | 1.78 (1.05–3.01) | 0.032 |
| SPE (STCL/SPCL) | 0.57 (0.34–0.93) | 0.025 |

*Model: n=85, events=42, EPV=21. Events-per-variable = 21 (≥10), so the two-predictor model is not overfitted. Collinearity between PHI and SPE: Pearson r = -0.264 (p = 0.015); VIF = 1.07 — well below any concerning threshold (VIF < 5), so both may enter the model simultaneously. Functional patella alta (binary) vs instability: OR 10.25 (95% CI 2.16–48.66), p = 0.003.*

**5c. Age-adjusted models (each predictor + age, per 1 SD)**

| **Predictor** | **Predictor OR (95% CI)** | **p** | **Age OR (95% CI)** | **p (age)** |
| --- | --- | --- | --- | --- |
| PHI (PTD/SPCL) | 1.65 (0.95–2.88) | 0.077 | 2.86 (1.59–5.17) | <0.001 |
| SPE (STCL/SPCL) | 0.62 (0.37–1.06) | 0.079 | 2.85 (1.57–5.16) | <0.001 |
| PTD (mm) | 2.84 (1.35–5.95) | 0.006 | 2.14 (1.13–4.06) | 0.019 |
| PTL (mm) | 3.11 (1.50–6.46) | 0.002 | 2.03 (1.06–3.89) | 0.032 |
| TT–TG (mm) | 3.56 (1.73–7.32) | <0.001 | 1.96 (1.03–3.74) | 0.041 |

*KEY FINDING: after adjustment for age, PHI (p=0.077) and SPE (p=0.079) are no longer statistically significant, whereas PTD, PTL and TT–TG remain independently associated with instability. Because the groups are not age-matched and age is itself strongly associated with OPI, age is a confounder for the PHI/SPE association. This has direct implications for the manuscript's central claim and requires an explicit decision by the author team (see accompanying summary).*
