# Supplementary material for: Increased sagittal patellar height and reduced trochlear engagement on MRI are associated with recurrent patellar instability in skeletally immature knees: A comparative study
Source: J Exp Orthop. 2026 Aug 21;13(3):e70896. doi: 10.1002/jeo2.70896 (PMC13495771; doi:10.1002/jeo2.70896)
Supplement: Supplementary file 2 [file JEO2-13-e70896-s003.docx]

**Supplementary Table S1. Age-adjusted logistic regression for objective patellar instability**

Sensitivity analysis. Each MRI parameter was entered in a logistic regression model together with age; odds ratios for continuous predictors are expressed per one standard deviation increase (n = 85 knees, 42 events).

| **Predictor** | **Predictor OR (95% CI) per SD** | **p** | **Age OR (95% CI) per SD** | **p (age)** |
| --- | --- | --- | --- | --- |
| PHI (PTD/SPCL) | 1.65 (0.95–2.88) | 0.077 | 2.86 (1.59–5.17) | <0.001 |
| SPE (STCL/SPCL) | 0.62 (0.37–1.06) | 0.079 | 2.85 (1.57–5.16) | <0.001 |
| PTD (mm) | 2.84 (1.35–5.95) | 0.006 | 2.14 (1.13–4.06) | 0.019 |
| PTL (mm) | 3.11 (1.50–6.46) | 0.002 | 2.03 (1.06–3.89) | 0.032 |
| TT–TG (mm) | 3.56 (1.73–7.32) | <0.001 | 1.96 (1.03–3.74) | 0.041 |

*After adjustment for age, the associations of PHI and SPE with objective patellar instability were attenuated and no longer reached statistical significance, whereas PTD, PTL and TT–TG distance remained independently associated with instability. As the two groups were not matched for age, and as patellar height and trochlear morphology both evolve during skeletal growth, age is a plausible confounder of the associations observed for the ratio-based indices. PHI = patellar height index; SPE = sagittal patellofemoral engagement; PTD = patello-tibial distance; PTL = patellar tendon length; TT–TG = tibial tubercle–trochlear groove.*
